# Protocol for a scoping review examining the application of large language models in healthcare education and public health learning spaces

**DOI:** 10.1371/journal.pone.0339594

**Published:** 2026-01-02

**Authors:** Henry Ndukwe, Emmanuel Oloche Otukpa

**Affiliations:** 1 School of Pharmacy and Medical Sciences, Griffith University, Brisbane, Australia; 2 Health and Wellbeing Theme, African Population Health Research Center (APHRC), Nairobi, Kenya; South China Normal University, CHINA

## Abstract

**Objective:**

Through this scoping review, we aim to explore and synthesize existing knowledge and evidence on the learning approaches for incorporating LLMs into healthcare education and public health research and learning spaces. Specifically, we will attempt to investigate methods for auditing prompts for accuracy, fairness, and effectiveness; tailoring prompts to improve task-specific accuracy and utility; and exploring how end-user feedback is used to refine and optimize LLM prompts over time. This review will provide a comprehensive understanding of how LLMs are being tailored and improved in these fields, contributing to the development of evidence-based strategies for their implementation. It will also identify areas for future research and innovation.

**Introduction:**

The increasing integration of large language models (LLMs) into healthcare education and public health research and learning spaces, highlights their potential to revolutionize service delivery, decision-making, and ultimately patient care and outcomes. Despite these advancements, understanding how LLMs can be effectively tailored, audited, and refined for learning remains a critical area of inquiry. Key issues include, the accuracy of generated information, and their relevance to the medical and public health fields.

**Inclusion criteria:**

Our focus will be on studies addressing LLM applications in healthcare education and public health research and learning spaces, prompt engineering techniques, prompt auditing methods, and processes geared towards integrating user feedback. Articles that do not focus on healthcare or public health contexts and lack relevance to LLM learning approaches will be excluded.

**Methods:**

The review is guided by the JBI methodology for scoping reviews complemented by updates from Levac et al. Databases including PubMed, Scopus, IEEE Xplore, and Web of Science will be searched for peer-reviewed articles, conference proceedings, and grey literature published in English and French from 2015 to 2025. Data extraction will include information on study characteristics, LLM models, prompt engineering strategies, auditing methodologies, and user feedback mechanisms. We will synthesize to identify trends, gaps, and best practices in leveraging LLMs to generate baseline data for auditing prompts that optimize AI learning and education needs in the healthcare and public health sector.

## Introduction

### Background and context

Large language models (LLMs) represent a novel innovation in computing and the part of what constitutes generative artificial intelligence, offering the potential to revolutionize healthcare and public health through its understanding of human language, terms and terminologies as well as its text generation capabilities [1]. Rooted in the concept of Deep Learning (DL), LLMs represent the latest subtype of artificial intelligence whose purpose is to generate new text, audio, video or any other type of content from a corpus of existing training data [2]. Essentially generating new data that has similar characteristics as data from the original dataset [2,3].

The historical foundations of generative AI and by extension LLMs are focused on the mathematical probabilities that explore the ability of computers to think like humans [4]. The development of the Bayesian Neural Network by Judea Pearl in 1985 and the recurrent neural networks by Irwin in 1986 serve as pivotal starting points in the growth of LLMs [2].

Additionally, the base template of an LLM as suggested by Bengio and colleagues through techniques for modelling a language uses a feed-forward neural networks [5]. At the heart of the problem in modeling a language is the concept of dimensionality [4]. While the dimensions of a problem in calculating statistical probabilities to reach an outcome or calculating the areas and spaces of geometry are known, the joint distribution of consecutive words in a natural language with an evolving vocabulary becomes exponentially more difficult to predict [4]. A simplistic view of an LLM is as a statistical model trained on a large amount of information. LLMs predict language patterns relatively accurately by using a structure loosely inspired by the neural network of a brain.

The constant and ever-changing corpus of information to be trained on these statistical models necessitated an additional tool for efficient learning [6]. LLMs fully came to their own when the concept of a *Transformer* was introduced [6,7]. Transformer architecture is a very efficient method for statistical modelling of unlabeled data centered around a concept known as self-attention [8]. These models, exemplified by Generative Pre-trained Transformers (GPT) and its counterpart, the Bidirectional Encoder Representations from Transformers (BERT) [6–8], can process and generate human-like text, finding applications in diverse industries. The generative component of LLMs lie in the predictive probabilities borne of the corpus of data it has been trained on [8].

In healthcare; clinical decision-making, patient education, administrative efficiency, and medical and clinical education and public health research constitute a large corpus of information representing LLM training data [9,10]. As healthcare systems increasingly explore digital solutions to enhance care delivery, the role of LLMs is becoming more prominent. Particularly in the learning and education of health providers across different spheres of practice. However, maximizing their potential while ensuring reliability and safety requires a nuanced understanding of their integration into healthcare-specific contexts [11].

The integration of LLMs into healthcare education and public health research and learning spaces presents opportunities and challenges. On one hand, LLMs offer unprecedented capabilities for personalized learning, real-time clinical decision support, and enhanced access to medical knowledge and public health data [12]. They can assist in creating customized educational content, simulating clinical scenarios, and providing immediate feedback to learners using artificial intelligence (AI) models. And on the other hand, LLMs may help address the growing burden of healthcare-information overload and inaccurate online searching by synthesizing dense and complex public and medical health information to relatively accurate and digestible output formats [11,12].

There is a growing body of literature that has explored the use of LLMs in healthcare education and public health research and learning. In 2023 Alaa Abd-alrazaq and colleagues noted the advances of the previous iteration of GPT-4, highlighting its potential of changing medical education in areas like curriculum development, teaching methodologies, personalized study plans and assessment for students. In addition to creating clinical case studies, simulating virtual patients and facilitating research [13]. The authors, however, noted key issues such as misinformation and lack of reliability and consistency, overreliance and algorithmic bias as challenges in its use.

Similarly, Safranek et al also in 2023, while exploring the role of LLMs in medical education acknowledged their benefits across different use-cases. The authors noted that the changing landscape puts the onus on the student in discerning the strengths and weaknesses of LLMs noting the legal and ethical issues abound in its use [14].

In nursing education, LLMs offer tailored care recommendations, medication management support, workflow automation with nursing records, and emotional support for patients. They are particularly valuable in rural or remote settings for timely advice and patient assistance. Ethical concerns including patient privacy, technology overdependence, and maintaining human care authenticity remain critical considerations when implementing LLMs in nursing and public health education [15].

### Specific contextual use and applications of LLMs

LLMs are increasingly integrated into curriculum design processes by supporting the generation of learning objectives, alignment of content with competency frameworks, and rapid development of case scenarios. AI-assisted blueprinting has been shown to enhance coherence across health-professional curricula [16]. One practical and documented application come from researchers at the Harvard Medical School (HMS) Brigham and Women’s Hospital; who developed and tested a tool that uses generative AI to create learner-centric curricula fit for adoption to the wider medical faculty [17]. Initially targeted at students in form of a course module; the students first try to create curriculum content on their own, then work with generative AI systems to do the same thing [17]. Additionally, HMS have included a generative AI module into their training curriculum titled AI in Medicine (AIM) based on the evidence of GPTs performance in passing the U.S. Medical Licensing exams [17]. With the core objective of harnessing the power of LLMs’ knowledge synthesis capabilities in tandem with the exponential growth in medical knowledgebase, projected to have a net effect on student learning outcomes.

Recent studies indicate that LLM-enabled adaptive tutoring systems provide personalized explanations, formative feedback, and remediation tailored to learners’ needs. Such tools reduce educator workload while enhancing cognitive scaffolding in medical education [13]. Exemplified by Darthmouth and Stanford Medical Schools in the development of the NeuroBot TA ®, an AI teaching assistant based on Retrieval Augmented Generation (RAG) [18]. At its core, it is a generative AI that limits its learning capabilities to a private library comprising of a corpus of vetted information drawn from course content and learning materials which when following an LLM workflow, aims to reduce a student’s time effort in searching for and using appropriate learning materials [18].

In a different context, researchers at the University of Malaysia proposed a framework for the development of a personalized learning system using Bayesian network association rules and decision trees [19]. The personalized learning component they proposed adhered to the Meyer-Briggs Type Indicator in learning style pedagogical model applicable to medical and allied training and education [19,20].

In clinical simulations and skill enhancements, LLM-powered virtual patient simulations are increasingly used in Objective Structured Clinical Examinations (OSCEs). Voigt and colleagues in 2025 evaluated an LLM-based framework for enhanced clinical skills training through an application divided into a frontend and backend. Whilst the frontend provided user interface capabilities, the backend comprised of agents that mimicked the roles of patient, and, tutor [21]. The researchers were driven to go beyond a text-based dialogue for student interactions to include robotic patient simulations. Similarly, Sugamiya et al in 2019, with a focus on the scoring of medical and clinical skill, constructed a system consisting of a speech recognition module, and an automatic scoring system that checked against sample responses. Their system was evaluated by medical doctors [22].

Generative AI supports public-health education through outbreak scenario modelling, health-literacy training, and contextual translation of surveillance data for teaching. These systems help learners engage with real-world epidemiological decision-making [23]. A notable case of these centers around pandemic preparedness, i.e., in understanding how AI-driven epidemiological modeling is important in outbreak prevention vis-à-vis the shift to remote learning for public health training and medical education as illustrated by the COVID-19 pandemic [24]. There is a growing consensus on the need for policy formulation around the use of LLMs around the training public health workforce. Documented initiatives include the Duke Institute for health Innovation where medical students worked together with data experts to develop care-enhanced technologies for physicians. And University of Virginia Center for Engineering in Medicine involving medical students in the engineering labs to create innovative ideas in health care; among others [25].

Despite these potential benefits, several challenges arise when considering the incorporation of LLMs into healthcare and public health education. The traditional learning framework has a shifting focus from scarcity towards the ubiquity of information via LLMs; fostering trust that would ensure user safety on a continuous basis. Future strategies that optimize efficiency with the leverage of AI-driven tools would require adaptable mechanism of feedback and continuous improvement. Ethical concerns surrounding data privacy, bias in AI systems, and the risk of over-reliance on technology must be carefully addressed [11,13]. There are also questions around the quality and accuracy of information generated by LLMs, particularly in rapidly evolving (as well as niche) medical fields [13]. Furthermore, educators and policymakers face the task of developing appropriate frameworks for integrating LLMs into curricula for learning and teaching or clinical practice modules [13,14].

### Challenges and gaps in knowledge

Regardless of the promise of having a standard output scoring system for generative AI prompts, challenges remain in deploying LLMs effectively in healthcare education and public health learning spaces [26]. Key concerns include the accuracy of generated outputs, biases embedded in models, and their contextual relevance to specific healthcare needs [26,27]. Addressing these challenges necessitates the development of precise prompting and prompt engineering techniques to tailor LLM responses for specialized responses. Furthermore, auditing LLM outputs for accuracy, fairness, and effectiveness is critical to building trust and ensuring equitable healthcare delivery [28,29]. However, the methodologies and best practices for conducting such audits in healthcare and public health contexts are not well-documented [11,12].

### Role of user feedback

User feedback, particularly from healthcare and allied professionals undergoing continuous education, as well as students and learners who have been exposed to LLMs as a learning tool, plays a vital role in refining LLM performance [30]. Feedback mechanisms provide insights into how LLMs can better align clinical workflows and patient care priorities. While some studies have explored integrating user feedback in LLM applications, there remains a lack of clarity on how this process is operationalized and its impact on improving model outputs over time [28,30].

### Rationale for the scoping review

Given the rapid evolution of LLMs and their potential adoption in healthcare, a comprehensive synthesis of existing knowledge application is essential to understand ways for proper prompt engineering regarding generative AI. The ubiquitous nature of generative AI poses a persistent risk of giving misinformation for queries [31]. This is premised around the LLMs’ learning algorithms, which are built to optimize outputs based on the layered complexity of user input (prompt) [32,33]. It is crucial to understand possible oversight strategies to mitigate AI-information overload in learning and research environments. To that end, this review will attempt to explore how LLMs have been deployed and audited in healthcare education and public health learning spaces. By investigating the methods used to audit LLM prompts for strategies to enhance task-specific accuracy and utility, and the integration of user feedback can be proposed to refine LLM outputs for the learning use-case in healthcare, clinical and public health settings.

### Objectives

In this scoping review, we aim to explore the current state of LLM integration in two distinct contexts in healthcare education/practice and public health research and learning spaces, identify best practices, and highlight areas requiring further research and development. We seek to provide actionable insights for researchers, healthcare professionals, and policymakers to optimize the use of LLMs in healthcare and public health education. The identification of best practices and knowledge gaps, it will contribute to advancing the safe and effective implementation of LLM technologies in these critical fields. Specifically, we intend to examine methods used to audit healthcare and public health LLM prompts for accuracy. Focused on prompts primarily oriented to the education of medical students and allied health professionals and public health as well as research learning spaces. Additionally, we will attempt to investigate how prompts are tailored to specific tasks to improve the accuracy, relevance, and overall utility of LLM-generated outputs. Finally, we intend to explore the integration of end-user (learner) feedback into the process of refining and optimizing LLM prompts over time, ensuring continuous improvement and alignment with real-world needs.

### Review questions

1. What methods are used to audit prompts for accuracy in healthcare and public health LLMs used in the training and education of health providers and public health researchers?
2. How are prompts tailored to specific tasks to improve the accuracy and utility of LLM outputs?
3. How have end-user feedback been integrated into the process of refining LLM prompts over time?

## Materials and methods

We intend to conduct this scoping review in accordance with the JBI methodology for scoping reviews [34]. The methodology entails a systematic approach to searching, screening, and reporting that include the following stages: (1) identification of the research question (s); (2) identification of relevant databases and studies; (3) selection of studies; (4) data extraction; (5) interpretation, summarization and dissemination of the results.

### Inclusion criteria

We intend to focus on studies whose primary lens is on the application or development of LLM usage and integration in healthcare or public health learning and education contexts. Examples of relevant contexts include clinical decision support, patient education, administrative processes, and public health interventions and education modules. In terms of the scope of LLM integration, the studies must explore learning approaches for integrating LLMs, including prompt engineering, auditing methodologies, and user feedback mechanisms.

Specific publication inclusion criteria will focus on peer-reviewed articles, conference proceedings, and grey literature (e.g., reports, white papers). Additionally, all study designs are eligible, including experimental, observational, qualitative, and mixed-methods studies. Studies focusing on frameworks, methodologies, and case studies will also be considered. We will also consider any study published in English or French language. And with a timeframe for inclusion from 2015 to 2025, reflecting a period of rapid advancements in LLM technology and its applications.

In terms of relevance, studies must address at least one of the following core areas:

- Methods for auditing LLM prompt for accuracy, fairness, and effectiveness.
- Techniques for tailoring LLM prompts to healthcare-specific tasks/learning outputs.
- Approaches for integrating user feedback from healthcare professionals to refine and improve LLM outputs.

### Search strategy

Our search strategy will aim to locate both published and unpublished studies. A three-step search strategy will be utilized in this review. First, we will conduct an initial limited search of MEDLINE (PubMed) and CINAHL (EBSCO) to identify articles on the topic. The text words contained in the titles and abstracts of relevant articles, and the index terms used to describe the articles will be used to develop a full search strategy for reporting the name of the relevant databases/information sources (S1 Table). The search strategy, including all identified keywords and index terms, will be adapted for each included database and/or information source. The reference list of all included sources of evidence will be screened for additional studies. We will search relevant peer-reviewed, English and French-language articles published between January 1, 2015, and January 31, 2025, without methodological restrictions, in several electronic databases, as well as sources with broad specificity (Web of Science and Google Scholar).

### Source of evidence selection (databases)

We will include varied electronic data sources including PubMed, Web of Science, CINAHL for nursing and allied health literature, ERIC for educational research, PsycINFO to capture behavioral and psychological aspects of LLM integration, Google Scholar and IEEE Xplore. Search will include databases for bibliographic sources including Medline, Embase, and Scopus. Additionally, we will also extend our searches to grey literature sources, including institutional reports, white papers, and preprints on platforms like arXiv, and medRxiv.

### Search terms

We will employ adjacency search and combination of keywords and Medical Subject Headings (MeSH) terms will be used, including: “large language models*”[tiab], “prompt engineering”[tiab], “healthcare”[MeSH], “public health”[MeSH], “auditing methods”[tiab], “user feedback”[MeSH], “artificial intelligence”[tiab], “Artificial Intelligence”[MeSH]. The Boolean operators (AND, OR, NOT) will combine the above terms to refine quantity and quality of search hits. A detailed search strategy is provided in S1 Table.

### Study selection

We will use the online tool Covidence®, which allows for simultaneous title, abstract and full text article reviews. Two researchers will independently assess articles for inclusion by screening the titles, abstracts, and full texts of studies returned through the search process. Where there are disagreements between the two independent reviewers on the eligibility of a paper for inclusion, a third reviewer will adjudicate using same inclusion criteria to resolve the conflict.

### Data extraction

We will use a standardized data charting form (S2 Table) to extract relevant data from included studies. The following details will be extracted:

1. **Study Characteristics**: Title, authors, year, and country of publication.
2. **LLM Details**: Type of LLM, application context, and specific tasks addressed.
3. **Methodologies**: Techniques for prompt engineering, auditing methods, and feedback mechanisms.
4. **Outcomes**: Measures of effectiveness, fairness, and task-specific utility.
5. **User Feedback**: Processes for incorporating feedback from healthcare professionals and the impact on model refinement.

### Data synthesis and presentation

**Fig 1:**
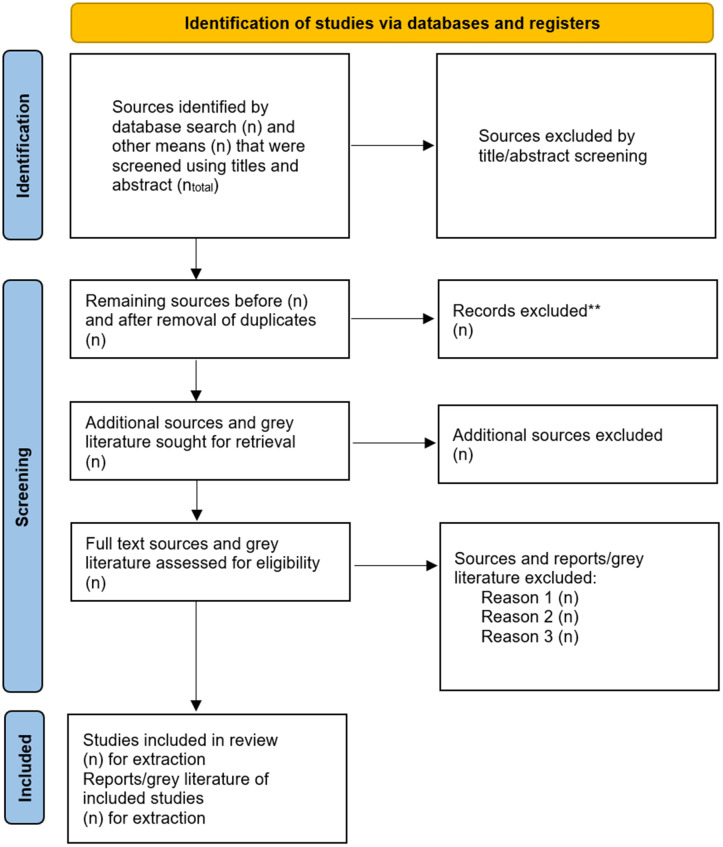
PRISMA flow diagram.

We will describe the literature search and study selection process using narrative synthesis and a visual PRISMA flow diagram (Fig 1) in accordance with the PRISMA Extension for Scoping Reviews recommendations [35], additionally we provide a PRISMA-P checklist as supporting information (S3 Table).

This figure illustrates the study selection process for the scoping review, detailing the logical sequence of records that will be identified, included, and excluded at each stage.

We will analyze the data using descriptive statistics and thematic analysis, with results organized in tables and charts and presented into themes that reflect the review objectives. Our thematic analysis will identify patterns and trends in the application of LLMs. Quantitative data, where applicable, will be summarized descriptively. We will also conduct a narrative synthesis to integrate findings across studies, focusing on methods, outcomes, and identified gaps.

### Ethics and dissemination

Ethical approval is not required because primary data collection is not involved in this study but rather analyzing both published and grey literature. However, the findings of this study will be disseminated through peer-reviewed publications and conferences as well as in relevant stakeholder forums. In case of any amendments to the protocol following its publication, we will provide the date of each amendment, describe the change(s), and report the rationale for the change(s) in future publications arising from this protocol.

### Study status and timeline

At the time of manuscript submission, this study is ongoing. Record screening is currently in progress and is expected to be completed within three weeks (by June 22, 2025). Data We will start data extraction will begin immediately after and is anticipated to. We anticipate that this exercise will be completed within an additional four weeks of finishing the screening phase (by July 20, 2025). We expect to have results ready for analysis and reporting by August 2025. Any deviations from this projected timeline will be documented and explained in future publications.

### Strengths and limitations

The strength of this review lies within the systematic approach to synthesizing the diverse evidence on the integration of large language models (LLMs) in healthcare, clinical and public health, with a focus on prompt engineering, auditing, and user feedback mechanisms, which is a relatively niche concept. By utilizing a broad range of sources, including peer-reviewed studies and grey literature, the review will provide a comprehensive understanding of current practices, trends, and gaps in the field. Its focus on healthcare-specific applications ensures relevance to real-world policy relevant challenges, while the inclusion of feedback mechanisms highlights its alignment with user-centered design principles. Conversely, potential limitations include the restriction to English and French language publications, which may exclude relevant studies in other languages, and reliance on available literature that may underrepresent unpublished or proprietary methods used by private collectives. Additionally, the rapidly evolving nature of LLM technologies means that findings may quickly become outdated, necessitating continuous updates to maintain relevance.

## Supporting information

S1 Table Search Strategy. (DOCX)

S2 Table Data Extraction Instrument. (DOCX)

S3 Table PRISMA-P Checklist. (DOCX)
