# Supplementary material for: Protocol for a scoping review examining the application of large language models in healthcare education and public health learning spaces
Source: PLoS One. 2026 Jan 2;21(1):e0339594. doi: 10.1371/journal.pone.0339594 (PMC12758804; doi:10.1371/journal.pone.0339594)
Supplement: S1 Table — (DOCX) [file pone.0339594.s001.docx]

# Appendix 1: Search strategy

# Database Search Strategy

We will use the advance search builder of the PubMed and CINAHL databases, which allows for the use of wildcards (*) and lengthy search terms. We limited our search to a period between 1^st^ January 2015 to 31^st^ January 2025 .

S1 Table. Search Strategy

| *#1* | *"Artificial Intelligence"[MeSH] OR "artificial intelligence"[tiab] OR AI[tiab]*  *OR "large language model*"[tiab] OR LLM[tiab]* |
| --- | --- |
| *#2* | *"Health Care"[MeSH] OR "Delivery of Health Care"[MeSH] OR healthcare[tiab]*  *OR "Public Health"[MeSH] OR "public health"[tiab] OR* |
| *#3* | *Education [MeSH] OR Research[tiab] OR “Learning spaces”[tiab] OR “Medical training*”[MeSH]* |
| *#4* | *"Audit"[MeSH] OR "Quality Assurance, Health Care"[MeSH]*  *OR audit*[tiab] OR auditing[tiab]* |
| *#5* | *"Student Feedback"[MeSH] OR "User Participation"[MeSH] OR "learner Satisfaction"[MeSH] OR feedback[tiab] OR "user feedback"[tiab]* |
| *#6* | *"prompt engineering"[tiab] OR prompting[tiab]* |
| *#7* | *#1 AND #2* |
| *#8* | *#7 AND #3* |
| *#9* | *#7 AND #3 AND #5* |
| *#10* | *#4 AND #6* |
| *#11* | *#8 AND #10* |
| *#12* | *#9 AND #10* |
