# Supplementary material for: Protocol for a scoping review examining the application of large language models in healthcare education and public health learning spaces
Source: PLoS One. 2026 Jan 2;21(1):e0339594. doi: 10.1371/journal.pone.0339594 (PMC12758804; doi:10.1371/journal.pone.0339594)
Supplement: S2 Table — (DOCX) [file pone.0339594.s002.docx]

# Appendix 2: Data extraction instrument

*S2 Table. Data extraction instrument*

| **Key domain** | **Data point** | **Description** |
| --- | --- | --- |
| Study Identification | Title | Indicates the study title |
|  | Authors | Indicates the names of the authors |
|  | Publication year | Indicates the year of publication |
|  | Country /region | Indicates the country of study and geographical region |
|  | Source | This indicates Database, journal, or organization where the study was found |
| Study characteristics | Study type | Type of study (e.g., experimental, observational, qualitative, case study) |
|  | Objective | Primary aim or research question of the study. |
|  | Context | Description of the healthcare or public health learning setting |
| LLM detail | LLM type | Name and version of the large language model used (e.g., GPT, BERT) |
|  | Application | Specific application of the LLM in healthcare or public health (e.g., clinical decision-making). |
|  | Task specific use | Specific tasks for which the LLM was used or tailored. |
| Prompt Engineering details | Techniques | Methods used to design and refine prompts for specific tasks. |
|  | Tailoring/Customizing strategies | Techniques for tailoring prompts to improve accuracy and utility. |
|  | Auditing process | Methods used to assess LLM outputs for accuracy, fairness, and effectiveness. |
|  | Auditing outcomes | Key findings or metrics from the auditing process. |
| End-user feedback | Feedback processes and mechanism | Processes for gathering feedback from healthcare professionals. |
|  | Feedback implementation | Description of how feedback was used to refine prompts or LLM outputs. |
|  | Feedback impact assessment | Outcomes resulting from feedback integration. |
| Results and outcomes | Key findings | Major findings relevant to the study objectives |
|  | Effectiveness | Measures of success or effectiveness of LLM application |
|  | Challenges | Any reported challenges or limitations in the study. |
| Gaps and relevance | Relevance to review objectives | Description of how the study aligns with the review objectives. |
|  | Identified gaps | Gaps or unanswered questions highlighted by the study. |
| Quality assessment | Randomized Trials/Interventions | Cochrane Risk of Bias assessment tool |
|  | Observational studies | Newcastle-Ottawa scale (NOS) for non-randomized studies |
|  | Qualitative studies | JBI Critical Appraisal Checklist |
|  | Study limitations | Limitations or biases reported by the study authors |
|  | Reviewer observations | Any additional domains or observations identified by the reviewer. |
